# Magnetoencephalography resting-state correlates of executive and language components of verbal fluency

**DOI:** 10.1038/s41598-021-03829-0

**Published:** 2022-01-10

**Authors:** Victor Oswald, Younes Zerouali, Aubrée Boulet-Craig, Maja Krajinovic, Caroline Laverdière, Daniel Sinnett, Pierre Jolicoeur, Sarah Lippé, Karim Jerbi, Philippe Robaey

**Affiliations:** 1 Service Hématologie-Oncologie, Charles-Bruneau Cancer Center, Sainte-Justine Hospital, Montreal, QC Canada; 2 Department of Neuroscience, Faculty of Medicine, University of Montreal, Montreal, QC Canada; 3 Department of Biomedical Engineering, École Polytechnique de Montréal, Montreal, QC Canada; 4 Department of Neurology, CHU Notre-Dame Research Center, University of Montreal, Montreal, QC Canada; 5 Department of Psychology, University of Montreal, Montreal, QC Canada; 6 Department of Pediatric, CHU Sainte-Justine Research Center, Montreal, QC Canada; 7 Department de Psychiatry, University of Montreal, Montreal, QC Canada; 8 Children’s Hospital of Eastern Ontario, Ottawa, ON Canada; 9 Department de Psychiatry, University of Ottawa, Ottawa, ON Canada

**Keywords:** Cognitive control, Language

## Abstract

Verbal fluency (VF) is a heterogeneous cognitive function that requires executive as well as language abilities. The purpose of this study was to elucidate the specificity of the resting state MEG correlates of the executive and language components. To this end, we administered a VF test, another verbal test (Vocabulary), and another executive test (Trail Making Test), and we recorded 5-min eyes-open resting-state MEG data in 28 healthy participants. We used source-reconstructed spectral power estimates to compute correlation/anticorrelation MEG clusters with the performance at each test, as well as with the advantage in performance between tests, across individuals using cluster-level statistics in the standard frequency bands. By obtaining conjunction clusters between verbal fluency scores and factor loading obtained for verbal fluency and each of the two other tests, we showed a core of slow clusters (delta to beta) localized in the right hemisphere, in adjacent parts of the premotor, pre-central and post-central cortex in the mid-lateral regions related to executive monitoring. We also found slow parietal clusters bilaterally and a cluster in the gamma 2 and 3 bands in the left inferior frontal gyrus likely associated with phonological processing involved in verbal fluency.

## Introduction

One way to evaluate executive functions is through verbal fluency (VF) testing. In a Verbal Fluency Letter (VFL) test, the participant is instructed to name as many words as possible starting with specific letters (F, A, and S in English), while following several binding instructions. VF is a test widely used in the clinic in order to detect executive impairment, for example to document deficits following traumatic brain injury^1^ or neurotoxic treatment in oncology^2^, focal cortical lesions^3^, and disease progression in different forms of dementia^4–6^. Among all executive functions, cognitive flexibility, inhibition, and processing speed are the best predictors of verbal fluency performance^7^.

Executive functions are high-level cognitive processes that control lower-level processes in the service of goal-directed behavior. They include abilities such as response inhibition, interference control, working memory updating, and set shifting. Based on the factorization of different behavioral tests, the unity and diversity framework describes a common factor related to inhibition and specific factors, such as updating and switching^8–10^. Executive functioning is often associated with the frontal lobes. A meta-analysis found better executive functions was associated with larger volume and greater thickness in prefrontal cortex^11^. Functional neural correlates of executive functioning revealed by another meta-analysis has shown a common pattern of activation in the prefrontal, dorsal anterior cingulate, and parietal cortices across executive function domains^12^. In general, executive tests appear to be sensitive but not specific for measuring frontal lobe functioning. In other words, other brain regions are needed to perform executive tests. One explanation for this contrast is that executive tests are complex and combine high-level control processes with lower-level non-executive processes that they control. Impairment of these lower-level processes will also affect performance on executive tests. Thus, frontal lobe involvement in virtually any executive process is probably a necessary but insufficient condition for optimal performance^13^.

With regard to VF tests in particular, previous neuroimaging and lesioning studies have suggested that they primarily reflect the frontal cortex functioning^6,14^. Prefrontal cortex volume was correlated with VF in a meta-analysis^11^. A meta-analysis of coordinate-based activation likelihood estimation (ALE) of brain activation during VF tasks in healthy volunteers showed that the main activation clusters are found in left frontal cortex inferior/middle gyri (BA 6, 9, 44 and 45) and right frontal lobe (BA 44, 47) the left precuneus (BA 7), as well as in bilateral insula (BA 13) and anterior cingulate gyrus (BA 24, 32)^15^. Lesions or perturbation studies have reinforced this view. Phonemic word fluency was more severely impaired in patients with lesions in the right frontal lobe than in the left frontal lobe, but lesions in both frontal lobes have shown significant decrease in fluency performance compared to control^16^. Cortical modulation excitability of the left dorsolateral prefrontal cortex via transcranial direct current stimulation (tDCS) showed a significant increase in the number of words produced after a letter cue^17^. Thus, performance in VFL shows great sensitivity to frontal lobe damage and executive functions impairment; this sensitivity is only slightly lower than that of the Wisconsin Card Sorting Task, which is a typical test for executive functions^13^.

However, as every executive function test, VFL is a hybrid test, due to its verbal nature. Regression analyses clearly demonstrated the hybrid nature of verbal fluency tests by showing that both executive control abilities (such as working memory updating and inhibition) and verbal abilities (such as vocabulary or lexical access) explained the number of words produced in fluency tasks^18^. In another study, scores from fluency tasks were entered into an exploratory factorial analysis with the scores of language tests (WAIS Vocabulary subtest and Boston Naming Test) and executive functioning tests (Wisconsin Card-Sorting Test and Trail-Making Test Part B). A two-factor solution was obtained: language tests logically loaded on the language factor, and executive tests loaded on the other executive factor. However, scores of verbal fluency loaded exclusively on the language factor^19^. Among the frontal brain neural correlate of VF, the left inferior frontal gyrus (BA 44, 45) as well as the left premotor cortex (BA 6) are involved in speech production through the well establish language functional network^20,21^. This raises the question of whether the frontal lobe functional correlates of verbal fluency are attributed to language or executive function. This question especially arises for the left hemisphere, where language is dominant.

One of the main limitations in studying brain activity of the executive and language component of VF is the difficulty of recording this brain activity while the participants names words. An overt approach produces motion artifacts, whereas a covert approach does not allow for assessment of task performance. It is therefore difficult to generalize conclusions between the overt and covert paradigms. Moreover, the cognitive processes may be different in the two approaches. The same difficulty arises for the baseline condition, which can vary considerably from overt or covert word repetition to a resting state. For this reason, we turned to the resting state magnetoencephalography (MEG) correlation cluster analysis method, as we had previously done on the same group of subjects to map the neural correlates of working memory in the resting state^22^. The objective of this study was to distinguish clusters associated with language or executive components of VF on the basis of correlations between MEG activity and performance on executive and/or language tests. In the present study, we first map the resting state neural correlates of Verbal Fluency Letter (VFL) scores. Then we entered the VFL and another neuropsychological test scores into a factor analysis to extract a factor that measures a relative advantage for VLF over this other test. Specifically, we measured the relative advantage for VFL over another verbal test (Vocabulary-VOC), and over another executive test (Trail Making Test-Condition 4-TMT). Using conjunction maps, we identified which clusters among those initially obtained for VFL were associated with better performance for VFL compared to VOC, or compared to TMT, or compared to both. This method using relative comparison with two neuropsychological tests allowed us identifying more specific clusters of VFL. We hypothesize that these correlation clusters could separate executive-related processes from speech production processes.

## Method

### Participants

Twenty-eight healthy subjects (13 males and 15 females, 25.76 ± 4.84 years old) with no reported history of neurological or psychiatric disorders took part in this study. The project was reviewed and approved by the University of Montreal and the CHU Sainte-Justine Research Ethics Board. Informed consent was obtained before the experiment and financial compensation was given upon completion of the experiment. We confirm that all experiments were performed in accordance with relevant guidelines and regulations.

### Neuropsychological assessment

A neuropsychological evaluation was carried out the same day as the MEG recordings. For verbal fluency, we used the Verbal Fluency-letter (VFL) subtest from the Delis-Kaplan Executive Function System^23^. In this test, participants are asked to generate as many words as possible for 60 s beginning with a given letter. This test assesses the ability to rapidly generate words by letter, following specific rules: words cannot be repeated, cannot be names or people or places, numbers, or grammatical variants of previous responses. To assess verbal abilities, we used the Vocabulary subtests (VOC) from the Wechsler Adult Intelligence Scale—4th Edition^24^. In the Vocabulary subtest, participants have to define up to thirty words presented orally by the tester. Participants can define the given word by using synonyms, by its use, a general category to which the word belongs, a clear or primary characteristic, some concrete examples of action or causal relationship. Each word is scored 2, 1 or 0, depending on the degree of comprehension of the word as well as the precision and the clarity of the response. As executive functioning test, we used the Trail Making Test Condition 4 (TMT) from the Delis-Kaplan Executive Function System Trail Making Test^23^. In the condition 4, participants have to draw lines alternating between letters and numbers printed on a page, but in their alphabetical or numerical order as quickly and accurately as they can. The response is scored as completed within the time limit. In all the analyses, we only used the scaled scores, corrected for age. The three test scores did not show any significant correlation between them: VFL and TMT (r = − 0.013; p = 0.947), VFL and VOC (r = 0.211; p = 0.282), and TMT with VOC (r = 0.350; p = 0.068).

The following 5 sections have already been described in^22^, but we have reproduced and adapt them below so that the reader can find all the necessary information in this paper.

### MEG and anatomical MRI data acquisition

All 28 subjects were comfortably seated with eyes open, fixating a back-illuminated screen located 75 cm in front of them. Two 5-min periods of resting state were recorded at a sampling rate of 1200 Hz, using a CTF-VSM whole head 275-sensor MEG system equipped SQUID detectors (superconducting Quantum Interferences Devices) in MEG core facility, Psychology Department, University of Montreal, QC, Canada. Following standard procedures, third-order gradiometer noise reduction was computed based on twenty-nine reference channels. Bipolar EOG (Vertical EOG and Horizontal EOG) was recorded in order to monitor eye blinks and ocular movements. ECG was also recorded to monitor heartbeats. Three head coils fixed at the nasion and the bilateral preauricular points were used for head localization and were monitored at the beginning and the end of each session. Particular care was taken to ensure that head displacement across sessions remained below 5 mm. The neuropsychological assessments were done in the morning at the Ste-Justine Hospital (Montreal, QC, Canada). Later in the afternoon, the participants went to the MEG facility, located in the Psychology Department of the University of Montreal, for the MEG recordings. Structural MRI images were obtained for each subject with a 3-T General Electric (GE) scanner (Saint-Justine Hospital, Montreal, QC, Canada). The individual surfaces were used to carry out the co-registration between the MEG fiducial markers (LAP, NAS, RAP) and the MRI structural image. The exact position of the head was refined based on head shape position files obtained using a 3D-localization Polhemus system.

### Data pre-processing

MEG data pre-processing was performed using the Matlab-based Brainstorm open-source software (https://neuroimage.usc.edu/brainstorm/Introduction)^25^. The data was first notch filtered at 60 Hz, and then between 0.5 Hz and 120 Hz. Cardiac artefacts, eye blinks, and eye movements were corrected using the Signal-Space-Projection method (SSP)^26^. Fifty signal epochs, centred on each artefact, were selected, and a singular value decomposition was applied to each artefact using built-in Matlab functions. Eigenvectors explaining at least 10% of the variance of the artefacts were discarded and the remaining eigenvectors were used to define the SSP. The SSP method relies on a signal space decomposition procedure, where the statistical characteristics of the measured signals are used to determine the two subspaces spanned by the MEG brain signals and the unwanted artifacts, respectively. Projecting the continuous MEG data onto the signal subspace effectively removes the components belonging to the artifact subspace.

### MEG sources estimation

MEG source reconstruction was performed using a standard weighted minimum-norm approach, with the Brainstorm software^25^. T1-weighted brain volumes were acquired in all participants and were used to generate a cortical surface model, using the FreeSurfer software package v7.1.0 (https://surfer.nmr.mgh.harvard.edu)^27^. Forward modelling of the magnetic field was defined based on an overlapping-sphere method^28^. The weighted minimum norm solution was computed using a loose dipolar orientation constraint (set at 0.5), a signal-to-noise ratio of 3, whitening PCA and a depth weighting of 0.5^29^. The noise covariance matrix for each participant was estimated from a 2 min empty room recording performed earlier the same day (same acquisition parameters but with no subject in the shielded room) (e.g.,^30^). The source time series were initially reconstructed on a 15,000-vertex individual brain tessellation, and then spatially interpolated to the MNI ICBM152 brain template and down-sampled to a 10,000-vertices template.

### Spectral power analysis

Resting-state power spectral density (PSD) was measured using the modified Welch periodogram technique (1-s Hamming window and 50% time-window overlap). The mean PSD for each frequency band of interest was obtained for each subject by averaging the PSD values across the frequency bins of each band. Power estimates were computed for all elemental cortical sources and all participants in the following frequency bands: Delta (1–4 Hz), theta (4–8 Hz), alpha (8–13 Hz), beta (13–30 Hz), gamma 1 (30–60 Hz), gamma 2 (60–90 Hz) and gamma 3 (90–120 Hz). Next, for each participant, the PSD values (i.e. oscillatory power) were standardized using a z-score transformation (computed at each vertex using the mean and standard deviation of the power values across all vertices within the same frequency band).

### Correlation analyses and cluster-level statistics

A correlation analysis was carried out to probe the putative relationship across individuals between spontaneous brain oscillation power (separately for each frequency band) and neuropsychological tests and factors scores (see “Steps for conjunction analysis”).

The computation and assessment of correlation were achieved via a two-step procedure. First, the Pearson correlation coefficients were computed between resting-state power (z-scores) at each cortical vertex and scores from the neuropsychological tests. Next, the statistical significance of the correlation results was evaluated using t-statistics and nonparametric cluster-wise correction for multiple testing^31^. More specifically, the correlation was computed using MATLAB’s built-in *corrcoef* function at each cortical node (vertex), for each frequency band and each subject (n = 28).

In addition to computing the Pearson correlation coefficient, the correlation function also returns p-values obtained by transforming the correlation to create a t-statistic with n − 2 degrees of freedom. Setting the threshold for the first-level statistical significance to p < 0.05 (uncorrected) provides a spatial mask in source space.

Next, within this mask (i.e. significant correlation coefficients, uncorrected), we determined clusters of spatially contiguous (neighbouring) vertices, which were identified based on the FreeSurfer adjacency matrix. The correlation coefficients within each cluster were then added up, in order to obtain a cluster mass (cluster mass statistics).

To assess the statistical significance of the obtained clusters, we used nonparametric permutation testing. We randomly shuffled the subjects’ neuropsychological scores, while keeping the MEG source power data across subjects intact. This essentially creates random associations that destroy any putative correlation between the two types of observations. Using 1000 permutations of the data (and replicating the cluster mass computation described above for each set of permuted data) provides for an estimate for the null distribution against which we can then test the significance of the truly observed clusters in the original data.

Statistically significant correlation clusters at p_corr_ < 0.001 were then defined as those with a cluster mass larger than the correlation value ranked 999 on the null distribution. By choosing such a restrictive significance threshold (i.e. p < 0.001), not only do we minimize the type-I errors, but we also ensure that the reported results would remain significant—for instance, at a level of p < 0.05, accounting for up to 50 multiple tests—based on a regular Bonferroni correction. In other words, all our results remain significant at p < 0.05 if we correct for tests across all frequency bands, and all the WAIS-IV subtests and indices used (as the number of comparisons does not exceed 50).

The results reported here are therefore statistically significant at p < 0.001 levels, corrected across space, for each frequency band and neuropsychological test, but they are all also significant at p < 0.05 when corrected for comparisons across space, frequency bands, and subtests.

### Steps for conjunction analysis

**Figure 1:**
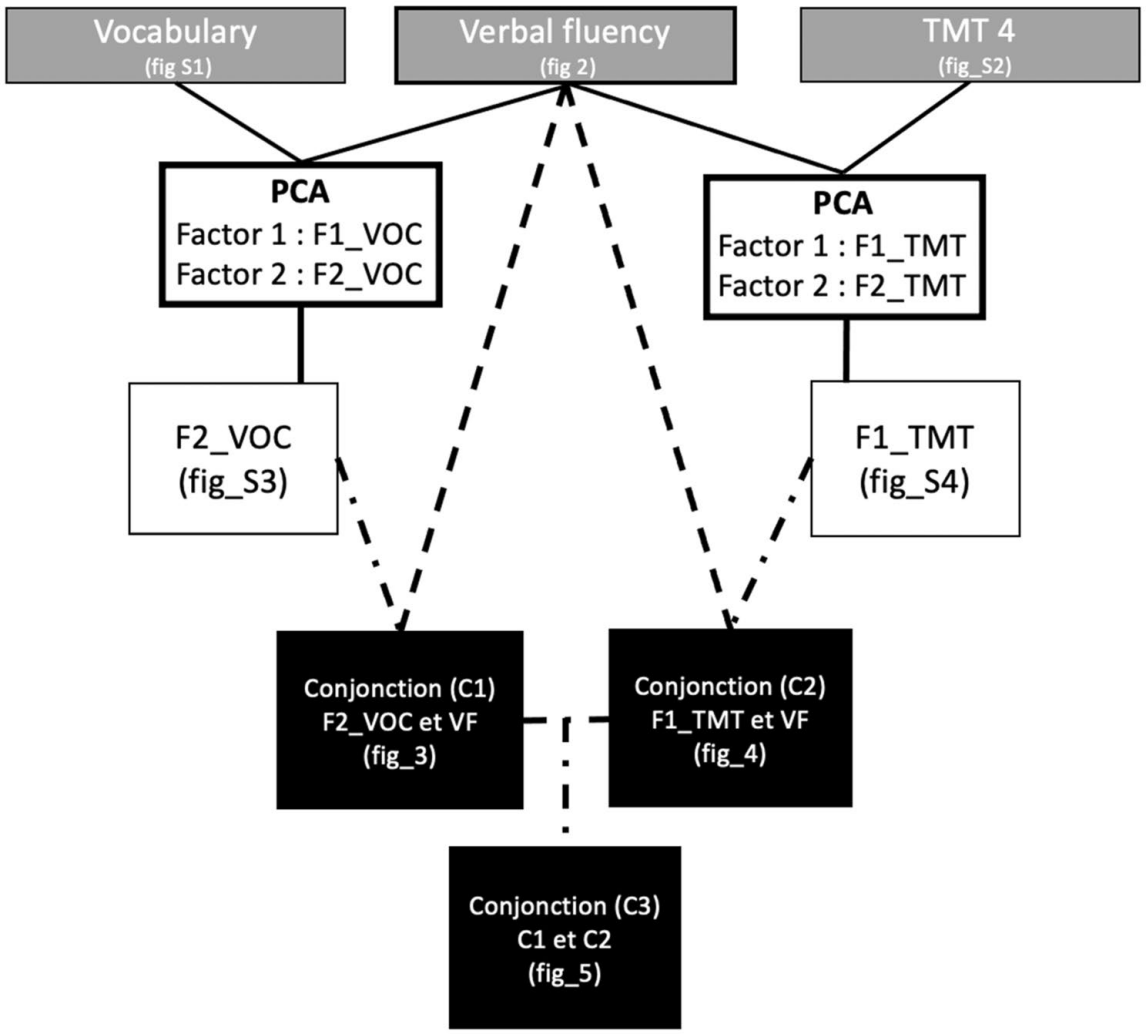
Schema showing the 3 steps methods used to explore relative specificity and different neural correlates of verbal fluency. The first step is to perform brain-behavior correlation/anticorrelation pattern between rsMEG power and each neuropsychological test independently (VOC, VF, TMT). The second step consists of factorizing Voc and VF and TMT and VF, from this factorization brain-behavior correlation/anticorrelation pattern was performed with residual regression from F2-VOC and F1-TMT. The third step was to make conjunction maps between F2-VOC & VF (conjunction 1) and F1-TMT & VF (conjunction 2). Conjunction 3 is the overlapping cluster between conjunction 1 & conjunction 2.

In order to differentiate verbal component and executive to the complex and sparse neural correlates of verbal fluency, we used a three-step method illustrated in Fig. 1.

#### Step 1: Cluster analyses with individual scaled tests scores

**Figure 2:**
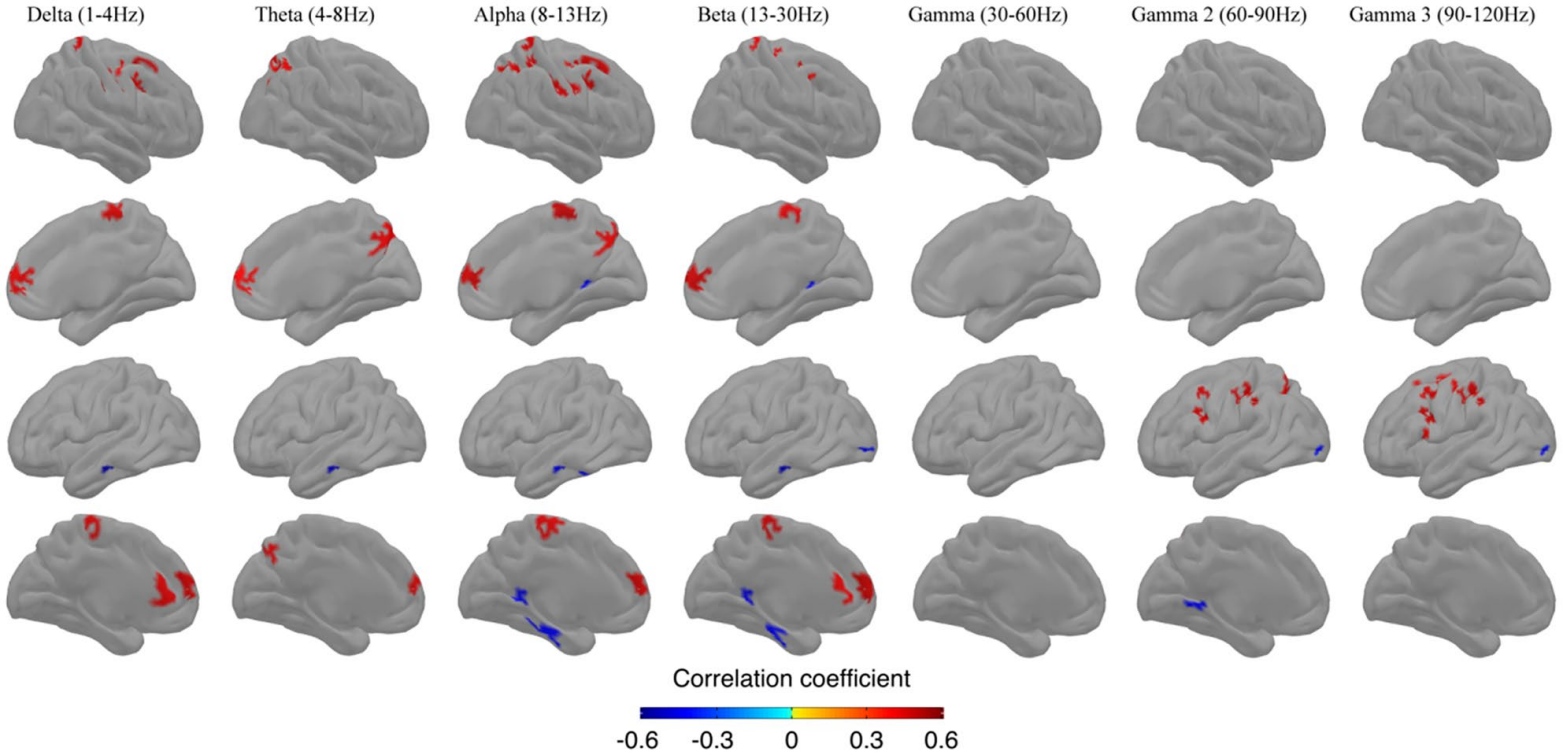
Group analysis (n = 28) spatial distribution of clusters with statistically significant correlations (p < 0.001) between resting MEG source-space power (z-scores across vertices) and neuropsychological performance on the Verbal Fluency test. Each column shows the significant correlations for a given range of frequencies, in both hemispheres (right lateral and medial view, followed by left lateral and medial views). The results are corrected across space using cluster-level corrections. All results remain significant at p < 0.05, correction for multiple comparisons across space and frequency bands.

This first step was to perform the cluster-level analysis of brain-test score correlation and anticorrelation patterns for each test (i.e. VFL, TMT and VOC). Figure 2 shows spatial distribution of correlation clusters between resting state source-space MEG power and scores on the VFL test; the correlation clusters obtained with VOC and TMT scores are presented in Supplementary material (Figs. S1 and S2 respectively).

#### Step 2: Cluster analyses with factor loadings combining test scores

Vocabulary knowledge is expected to play a role in the ability to produce words in a verbal fluency task because the subject has to activate and access the semantic knowledge network. For example, subjects with a larger vocabulary produced more words than those with a smaller vocabulary^32^, and children with Specific Language Impairment often showed deficits in VF performance^33^. Also, the vocabulary score correlated negatively with the first reaction time in a VFL task^18^. A higher score in the VFL than in the VOC should reflect a relative advantage in word production control over word knowledge, in other words, a relative advantage in non-semantic executive abilities over semantic non-executive abilities, in the language domain. We did not use differences between scores because they are relatively unreliable due to the correlation between them^34^. Instead, we decomposed the total variance of both tests using a principal component analysis (PCA), as the individual loadings are more reliable than averages or differences between scores^34^. By entering two test scores, a PCA accounts for the total variance with two factors. One factor was correlated with the average scores between the tests while the other factor was correlated with their difference. The first factor (F1-VOC) represented 60.5% of the total variance, while the second factor (F2-VOC) 39.5%. F1-VOC was highly and equally correlated with both the VFL and the VOC scaled scores (r = 0.778; p < 0.001), and with their mean scores (r = 0.928; p < 0.001). F2-VOC was also equally correlated with both tests, but in opposite directions: r = 0.628; p < 0.001 with VFL, and r = − 0.628; p < 0.001 with VOC. As a consequence, this factor was highly correlated with the difference between the VFL and VOC scores (r = 0.846; p < 0.001). Then we computed the MEG clusters with positive and negative correlations with the individual factor loadings on the difference factor (F2-VOC), which for positively correlated clusters reflect a better performance in VFL than in VOC. Results also presented in the Supplementary Fig. S3.

We used the same approach for VFL and TMT. In both tests, the subject had to produce and control a sequence of actions. In the VFL test, the subject had to generate a series of words while keeping them in memory and controlling that they followed a set of rules. However, the words did not have to follow a pre-ordered sequence. In contrast, in the TMT, the subject had to draw connecting lines between letters and numbers printed on a page, switching from letters to numbers and back, but exactly their alphabetical or numerical order. However, the subject did not have to remember the elements of the sequence that he or she could easily trace on the test page. A higher score in the VFL than in the TMT should reflect a relative advantage in monitoring items in memory and checking for compliance with a set of rules over attention to external cues using two overlearned orders and switching between them. Relative to TMT, the demand on working memory seems much larger in VFL. In this PCA, the first factor (F1-TMT) represented 50.6% of the total variance, while the second factor (F2-TMT) 49.3%. F1-TMT was equally correlated with both tests, but in opposite directions: r = 0.712; p < 0.001 with TMT, and r = − 0.712; p < 0.001 with VFL. As a result, F1-TMT was also correlated with the difference between VFL and TMT scores (r = 0.94; p < 0.001). F2-TMT was highly and equally correlated with both the VFL and the TMT scores (r = 0.702; p ˂ 0.001), and with their mean scores (r = 0.702; p ˂ 0.001). We computed the MEG clusters with positive and negative correlations with the individual factor loadings on the difference factor (F2-TMT), which for positively correlated clusters reflect a better performance in VFL than in TMT. Results also presented in the Supplementary Fig. S4.

In general, the clusters obtained for a difference factor are superimposed on the correlation clusters obtained for each of the two tests introduced in the factor analysis, one of them giving rise to anticorrelation clusters. The clusters associated with the difference factors generally involve more vertices, and more frequency bands, but the opposite is sometimes observed, which makes it possible to identify more specific clusters by computing conjunction maps.

#### Step 3: Conjunction analysis

In order to compare the pattern obtained with the two difference factors (F2-VOC and F1-TMT) and the initial pattern of VFL, the final and third step was to compute conjunction maps of cluster correlations.

**Figure 3:**
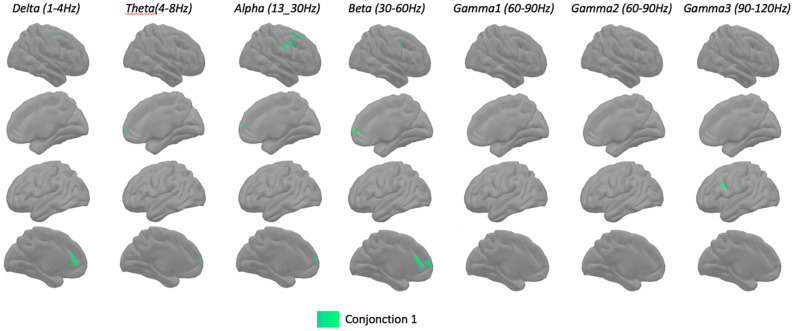
Group analysis (n = 28) for spatial distribution of clusters with statistically significant correlations (p < 0.001) between MEG source space power at rest (z-scores across peaks) and both F2-VOC and VFL scores (Conjunction 1). Each column shows the significant correlations for a given range of frequencies, in both hemispheres (right lateral and medial view, followed by left lateral and medial views). The results are corrected across space using cluster-level corrections. All results remain significant at p < 0.05, correction for multiple comparisons across space and frequency bands.

**Figure 4:**
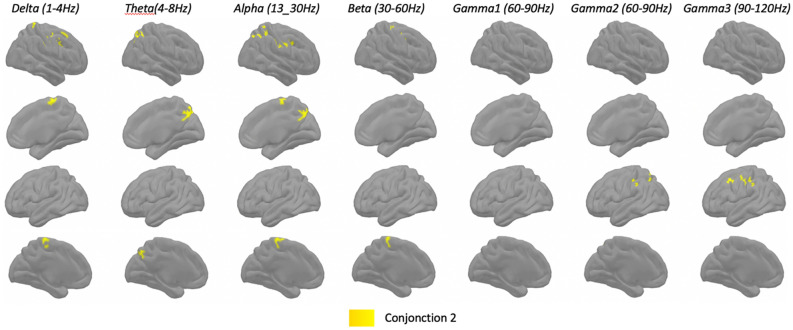
Group analysis (n = 28) for spatial distribution of clusters with statistically significant correlations (p < 0.001) between MEG source space power at rest (z-scores across peaks) and both F1-TMT and VFL scores (Conjunction 2). Each column shows the significant correlations for a given range of frequencies, in both hemispheres (right lateral and medial view, followed by left lateral and medial views). The results are corrected across space using cluster-level corrections. All results remain significant at p < 0.05, correction for multiple comparisons across space and frequency bands.

**Figure 5:**
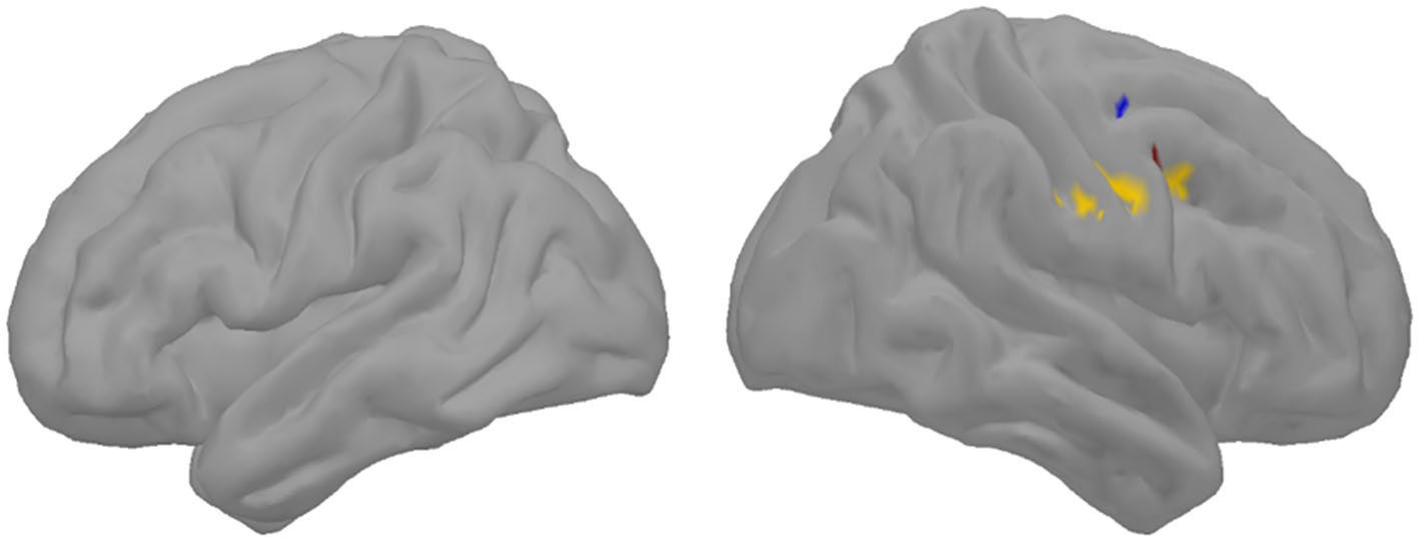
Group analysis (n = 28) spatial distribution of clusters with statistically significant correlations (p < 0.001) in both Conjunction 1 and 2 (Conjunction 3). Spatial frequencies are described as following: delta band (1–4 Hz) in blue, alpha band (8–13 Hz) in yellow, clusters found in both (alpha (8–13 Hz) band and beta (13–30 Hz) band) are in red.

To this end, we computed the overlap between each vertex (N = 10,000) across each frequencies band (N = 7). We computed three conjunction maps. For the first map (Conjunction 1), we retained the clusters from the initial VFL pattern that were both correlated positively with the individual factor loadings on the difference factor F2-VOC and with the VFL scores. These are the initial VFL correlation clusters that reflect a better performance in VFL than in VOC. For the second map (Conjunction 2), we retained the clusters from the initial VFL pattern that were both positively correlated with the individual factor loadings on the difference factor F1-TMT and with the VFL scores. These are the initial VFL correlation clusters that reflect a better performance in VFL than in TMT. For the third map (Conjunction 3), we computed the conjunction between conjunction 1 and conjunction 2. Conjunctions 1, 2 and 3 are presented in Figs. 3, 4, and 5, respectively.

## Results

### Verbal fluency letter

Figure 2 presents the clusters of behaviour neural correlation/anticorrelation with VFL. The lateral face shows correlation clusters in parietal, sensorimotor and frontal regions on the right for lower frequency bands (delta to beta range), as well as in the same regions on the left for high frequency bands (gamma 2 and 3 bands). Specifically, on the right lateral side, we found clusters in the superior parietal (delta to beta range); in the inferior parietal (theta band) lobe; in the postcentral and precentral gyri (delta, alpha and beta bands); and in the caudal part of the superior frontal (delta and alpha bands) as well in the caudal middle frontal gyri (delta, alpha and beta bands). On the left side, we detected clusters for high frequency bands only: in the superior parietal (gamma 2 band) and the supramarginal (gamma 2 and 3 bands); in the postcentral (gamma 2 and 3 bands) and precentral (gamma 3 band); and in the caudal middle frontal gyri (gamma 2 and 3 bands), in the caudal superior frontal and the pars opercularis (both in the gamma 3 band). On the medial hemispheres, the correlation clusters were mostly bilateral in the paracentral lobule (delta, alpha and beta bands), the precuneus (theta band), and the rostral superior frontal gyrus (delta to beta range). Additional clusters were observed in the left precuneus (theta band), in the left rostral anterior cingulate (delta and beta bands). Anticorrelation clusters were fewer and mostly found on the left temporal/occipital areas: in the middle temporal (delta to beta range), inferior temporal (alpha band) and lateral occipital (beta, gamma 1 and 2 bands) cortex. Medial anticorrelation clusters were also observed in the isthmus cingulate in the right (alpha and beta band) and left (alpha, beta and gamma 2 bands) hemispheres.

### Conjunction 1

Conjunction 1 (Fig. 3) shows the clusters common between the difference factor F2-VOC and the initial clusters for VFL. Three patterns emerged. First, some clusters were found in the right lateral hemisphere in the slow frequency bands, but predominantly in the alpha band. Specifically, they were found in the precentral and the caudal middle frontal in the delta, alpha and beta bands, in the post-central, precentral, caudal middle frontal and caudal superior frontal gyri (thus motor, premotor and dorsolateral prefrontal regions) in the alpha band. Small clusters were also present in the precentral and the caudal middle frontal gyri for the delta and beta bands. Second, small clusters were found bilaterally in the dorsomedial prefrontal cortex for the slow frequency bands, but they were larger on the left hemisphere in the alpha and delta bands. Specifically, clusters were found on the rostral and caudal anterior cingulate in the alpha and delta bands in the left hemisphere, as well as in the adjacent part of the medial superior frontal gyri from theta to beta bands on both hemispheres. Third, in gamma the 3 band, we found a cluster in the neighbouring precentral, caudal middle frontal and inferior frontal (pars opercularis) gyri on the left hemisphere.

### Conjunction 2

Conjunction 2 (Fig. 4) shows common clusters between the difference factor F1-TMT and the initial clusters for VFL. Again, three patterns emerged. First, we found clusters in the right post-central, pre-central and caudal middle frontal gyri for the delta, alpha and beta bands, as well as in the caudal superior frontal for the delta band only. Second, we found more posterior clusters, bilaterally in the precuneus for the theta band and in the paracentral lobule for the delta and alpha bands. Other clusters were observed in the right superior parietal lobule in the delta to alpha bands and in the left paracentral lobule for the theta band. Third, we found clusters on the left lateral hemisphere: in the postcentral and supramarginal gyri, and the superior parietal lobule for the gamma 2 band, and more anteriorly in the pre-central and caudal middle frontal gyri for the gamma 3 band.

### Conjunction 3

The result of conjunction 3 map analysis (Fig. 5) showed clusters only in the right hemisphere, in the ventral part of the pre- and post-central gyrus as well as in the adjacent caudal middle frontal gyrus in the alpha band. A cluster overlapping alpha and beta was also found in the precentral gyrus, and another cluster in the delta band was found more dorsally in the precentral gyrus.

## Discussion

The aim of this study was to provide the resting state MEG correlates of verbal fluency. Our results revealed a complex correlation pattern showing bilateral fronto-parietal clusters, right dominant in slow oscillations (delta to beta bands) and left dominant in highest oscillations frequency (gamma 2 and 3 bands). Anticorrelation clusters were also found in the left hemisphere temporal and occipital regions, in the right isthmus cingulate. Our results are in line with previous results report obtained during task-based approach of verbal fluency: similar activation was found in the left IFG/MFG (BA 6, 9, 44 & 45), the left precuneus (BA 7), and bilateral anterior cingulate gyrus (BA 24, 32). However, we did not find the right frontal lobe (BA 44, 47) nor the bilateral insula (BA 13) whereas we did find additional clusters in the bilateral motor cortex with paracentral lobule (BA 4), in the right premotor (BA 6, 8) and bilateral post central gyrus (BA 1, 2, 3)^15^. MEG fields are induced by synchronized neuronal currents, caused by synaptic transmission. Our results show that EEG/MEG power at rest may be correlated with performance during a test for different cortical sources and frequency bands.

The search for brain markers of cognitive traits has been recently boosted using brain global functioning markers. These markers have been showed to predict different phenotypes, including cognitive abilities. The most used brain marker is whole-brain MRI functional connectivity, based on the correlation over time of brain activity at rest in distinct regions. Functional connectivity can be viewed as “brain fingerprinting” allowing to identify an individual from a group of subjects. In addition, functional connectome fingerprinting has been used to predict cognitive individual differences by modelling the relationship between connectivity strength and task performance. This approach has been applied to predict fluid intelligence^35,36^, attention^37^, general cognitive ability^38^, or working memory^39^. On the other hand, it has been showed that a reliable brain fingerprint can be based not only on connectomes measures, but also much the simpler measures of the spatial distribution of spectral signal power of MEG activity recorded at rest^40^. Using MEG spectral fingerprinting we previously showed that the relationship between the spatial distribution of spectral signal power and working memory performance can be modeled in key medial and dorsolateral clusters within the parietal and prefrontal cortices^22^. Here we showed that power spectral fingerprinting can also be used to identify cortical clusters predicting executive functioning, and more specifically to differentiate the verbal and the executive component of verbal fluency test.

However, at the frequency band level, we do not have a mechanistic model of the relationship between the power of a particular frequency band of the resting MEG signal in a particular region and the performance in a task, or with the local power of oscillatory activity during the task itself. Some clusters were found for narrow frequency bands and could be related to the oscillatory phenomenon of power, while other clusters were found across broad frequency bands and could be related to the linear (1/f) part of the power spectrum. At the spatial level, we can assume that the cortical regions where signal strength predicts test performance overlap the regions which would be activated while performing the test itself. This assumption allows us to compare the spatial distribution of the clusters with an extensive task-based brain imaging literature. However, this assumption has limitations. One should not expect that all clusters from the resting state model would be activated during the same task. Resting-state MEG clusters are observable only to the extent that the relationship between interindividual differences in test scores and relative power at cortical sources can be modeled by a definite (e.g., linear) function. Conversely, all regions activated in a task are not expected to yield resting-state MEG clusters that predict performance in that task. The level of activity in specific brain regions during a task is not necessarily associated with the level of performance in the task. Moreover, in task-based brain imaging, specific activated regions are obtained by subtracting the activity in the task from a closely match control task. Resting-state correlation clusters are obtained using the performance in one or a set of tasks supposed to measure a cognitive trait but are not controlled for the performance in another task. In order to obtain more specific cluster, we must create new indices more specific than test scores, like standardized residual scores by regressing out another cognitive measure^22^, or factor scores opposing the performance between different tests, like in the present study. Resting state clusters may thus complement the task-based activity clusters by providing a more holistic view on regions involved in verbal fluency.

With conjunction 1, we aimed at identifying among the initial VFL clusters those accounting for a better performance in VFL as compared to VOC, thus for a relative advantage in executive over semantic abilities. A first group of clusters was found in the right premotor, motor and dorsolateral prefrontal cortex in the slow bands, most clearly in the alpha band. In early studies of executive function localization^41,42^, right lateral brain lesions, including the dorsolateral prefrontal cortex (BA9/46), thus overlapping some conjunction 1 clusters, had been linked to a deficit in monitoring ongoing performance in different executive tasks. This suggests that the relative advantage in executive abilities over semantic abilities is likely in monitoring verbal production. The second set of clusters was found in the dorsomedial prefrontal cortex, specifically in the bilateral rostral superior frontal and left rostral and caudal anterior cingulate, also in the slow frequency bands. This localization is consistent with lesions observed in patients whose performance deficits in various tasks have been described as an “energization failure”^41,42^. These patients showed lesions in superior medial cortex, primarily in BA areas 24, 32, 9, and 6. In the VFL task, they showed a marked decrease in the number of words in the last 45 s, compared with the first 15. This failure to energize is thus one of initiation and maintenance of performance. Moreover, these clusters are found in the alpha (8–13 Hz) and beta (13–30 Hz) oscillations, which are known to support inhibition mechanism^43,44^, which may be required to for maintenance of performance. These two clusters (right frontal lateral and dorsomedial frontal) correspond to the dual control network hypothesis^45^. The fronto-parietal network is optimized for rapid adaptive control and the other cingulo-opercular for stable set-maintenance. It also fits with the conjunction analyses across different executive functions (flexibility, inhibition and working memory), which also reveal activation in dorsolateral prefrontal (BAs 9, 46) and anterior cingulate (BA 32) cortex^12^. However, contrary to Wagner et al.’s^15^ previous meta-analysis, we did not find any parietal clusters in this conjunction 1.

The last set of clusters in conjunction 1 was found in the left inferior frontal gyrus (BA 44) in the gamma band. In phonemic and semantic verbal fluency tasks, activation in the left inferior frontal gyrus overlapped the same regions (BA 9, 45), but BA 47 appeared in the semantic condition, and BA 44 in the phonemic condition only^15^. This left inferior frontal gyrus was found in gamma band (90–120 Hz). Gamma band oscillations have been observed during word production and auditory perception. They reflect synchronized firing of neuronal assemblies and task-related cortical activation. Using electro-corticography (CoG), an increase in gamma range (70–120 Hz), activity was also observed continuously in the inferior frontal gyrus starting 500 ms prior to the onset of syllable-articulation and stopping at vocalization. The gamma-augmentation may thus be predominantly driven and/or monitored phonological processing^46^. The coexistence of gamma and alpha clusters in conjunction 1 in the left inferior frontal gyrus and right premotor, motor, and dorsolateral prefrontal cortex, respectively, may reflect a functional architecture between the left and right hemispheres through cross-frequency interactions between gamma and alpha activity^47^.

We first discussed, the conjunction 1 clusters that correlated positively both with the VFL scores and with the individual factor loadings on the difference factor and reflected an advantage in executive over semantic abilities. We also found clusters that were positively correlated with vocabulary scores (Fig. S1) but negatively correlated with individual factor loadings of the difference factor. They reflected an advantage of semantic abilities over executive abilities, (Fig. S3) and appeared to constitute a semantic knowledge network. These clusters were found for the low frequency bands in the left lateral temporo-parietal region: in the transverse, superior and middle temporal gyri, the inferior parietal and supramarginal gyri, the post-central gyrus as well as in the lateral occipital regions. According to the “embodied” view of semantic information, a word is associated with different representations of an object, for example how it looks like, or how it is used. The meaning of a word is then based on a network of visual, auditory, somato-motor representations. These widely distributed regions, and the various connections between them, constitute the semantic network^48–51^. Semantic representations can be based on different types of relations^52^. For example, similarity can use shared features (taxonomic, e.g. coat for dog-bear), or contiguity which relies on the co-occurrence in events or scenarios (thematic, e.g., dog—leash). By performing voxel-based lesion-symptom mapping on taxonomic and thematic errors separately in individual with poststroke aphasia, thematic errors were located in the left temporoparietal junction, and taxonomic errors in the left anterior temporal lobe^53^. The left temporo-parietal junction, where thematic knowledge has been proposed to be grounded, is the core of the F2 anticorrelation clusters we found. The temporo-parietal region (especially the posterior middle temporal cortex and the inferior parietal lobule) might be involved in mental simulation of events, or re-enactement of the subject’s own perceptual and motor experience. Using a picture matching task in which participants had to identify taxonomic and thematic relations between objects thematic processing specifically recruited a bilateral temporo-parietal network including the inferior parietal lobules and middle temporal gyri^54^. The clusters we found also include postcentral and occipital regions, which is consistent with the role of visual and sensorimotor regions in visuo-motor processes supporting thematic representations. In sum, the stronger the traces left by language experience in this thematic semantic network, the better the individuals perform in the Vocabulary test. Relying on the thematic semantic network would optimize the score at Vocabulary as participants can define the given word by using synonym, by its use, a clear characteristic, some concrete examples of action or causal relationship, and not necessarily by providing a general or more abstract category to which the word belongs.

Going back to the VFL correlation clusters, Conjunction 2 has two main differences from conjunction 1. The first difference was in slow frequencies, with bilateral clusters in the precuneus and paracentral lobule, and in the right superior parietal lobe. At the same time, clusters in the bilateral dorsomedial frontal cortex and in the right superior frontal gyrus disappeared. The second difference were additional gamma clusters in the left parietal, sensory-motor and premotor regions. These changes in resting state activity account for an advantage for VFL over TMT, as compared to VOC. VFL and TMT mainly assess two different executive functions, fluency, and flexibility, and differ mainly on two aspects. First, VFL requires the production of a verbal sequence, whereas TMT requires an eye-hand coordinated sequence. Second, in VFL the subject must keep in memory the entire verbal sequence as it is spoken to check that it conforms to a set of rules (first letter of the word, no repetition, etc.), but without following a pre-established order. On the other hand, in the TMT, the subject must follow a pre-established sequence (the alphabetical and numerical orders), and switch between them, but without keeping in memory the entire sequence since the test sheet provides a visual support. We suggest that the changes in predominantly right parietal slow oscillations may reflect the fact that the subject must pay attention to items in episodic memory (a form of working memory) during the fluency task. On the other hand, larger left frontal gamma clusters may be attributed to the speech production itself.

According to the attention-to-memory model, the dorsal parietal cortex is most active when top-down monitoring of memory content is maximal^55^. The dorsal parietal cortex corresponds approximately to BA7 and includes the superior parietal lobule, but also the precuneus and part of the paracental lobule. The additional parietal clusters in conjunction 2 may therefore correspond to top-down attentional processes in accordance with internal goals as per VFL instructions, and thus account for an advantage in performance in VFL over TMT, as compared to VOC. The presence of a medial parietal structure (precuneus) in the slow oscillation clusters is likely related to the fact that the task requires the subject to monitor his or her own verbal output, given that the precuneus supports the recall of memories from a first-person perspective^56,57^. The dorsal parietal cortex is part of a dorsal frontoparietal pathway that includes the midlateral prefrontal cortex and enables the selection of internal goals and links them to appropriate responses^58^. The absence of dorsomedial prefrontal clusters which were present in conjunction 1 may reflect the fact that the energization component of executive functioning is no longer an advantage for VFL when comparing with TMT over VOC, as initiation and maintenance of performance is greatly helped in TMT by the availability of the test sheet with the different letters and numbers to connect.

The rapid oscillatory cluster on the left corresponds with the word production sequence, from the left phonological store (i.e., left supramarginal gyrus) and then to pre-motor for the syllabification and ending in the pre-central gyrus for articulation^21^. Moreover, this cluster is found in the gamma bands, gamma 2 (60–90 Hz) and gamma 3 (90–120 Hz). As described previously, the gamma band were found in the left pre-central gyrus after onset of vocalization process^46^ and reflects local synchronized firing of neuronal assemblies.

Finally, the conjunction 3 (Fig. 5) shows the overlap between conjunction 1 and conjunction 2, highlighting a set of anterior frontal clusters related to the executive monitoring process and a set of more posterior frontal clusters related to phonological sequence implementation. Conjunction 3 thus reflects regions that must coordinate activity in order to perform in the fluency task. This overlap is localized in the right hemisphere, in adjacent parts of the premotor, pre-central and post-central cortex in the mid-lateral regions. Previous lesions study shown these regions are implicated in monitoring and control of speech production^41^.

To conclude, we first showed a complex set of correlation clusters, for the slow frequencies, in the right parietal and frontal regions, and in the bilateral medial frontal regions, bilateral paracentral and precuneus, as well as anticorrelation clusters in the left medial temporal lobe. By examining which of these clusters were related to a relative advantage in VFL as compared to two other tests (one verbal and one executive), we retained the core clusters of VFL. These core clusters clearly present verbal fluency as an executive test, related to word production and performance monitoring. Despite the verbal nature of verbal fluency, these clusters were located in the right hemisphere, which seems to reflect right-hemisphere dominance for executive control of attention^59^. This study confirms the value of the cluster analysis method based on correlations between resting MEG and cognitive skills, such as working memory or verbal fluency.

These results and this method require replication and further internal and external validation. The sample was small and susceptible to sampling bias effects. As MEG temporal dynamics mainly include oscillatory and non-oscillatory (1/f noise-like) brain activities, the nature of the correlation needs to be elucidated. Although we have observed correlation clusters for various cognitive abilities (working memory, verbal fluency, and vocabulary), it is not known which other cognitive skills may generate such clusters. It is also not known whether differences in performance outside the normal range could be detected by MEG clusters at rest, or whether other than linear functions should be used. This approach of cluster analysis is thus nascent, and requires much further research, but may have great potential to provide simple and holistic markers of cognitive functions.

## Supplementary Information

Supplementary Information.
