# Supplementary material for: Magnetoencephalography resting-state correlates of executive and language components of verbal fluency
Source: Sci Rep. 2022 Jan 10;12:476. doi: 10.1038/s41598-021-03829-0 (PMC8748602; doi:10.1038/s41598-021-03829-0)
Supplement: Supplementary file 1 — Supplementary Information. [file 41598_2021_3829_MOESM1_ESM.docx]

## Supplementary material

Supplementary Information:

### Vocabulary: Figure S1

Correlation clusters were found in neighboring areas in the parietal and temporal areas almost exclusively in the left hemisphere. In the parietal region, these clusters were localized in the postcentral (alpha to beta band), superior parietal (beta band), inferior parietal (theta to gamma 1 bands) and supramarginal (delta to gamma 1) gyri. In the temporal regions, we found clusters in the middle temporal (delta to gamma 1 and gamma 2), superior temporal (delta, theta and beta bands), transverse temporal (theta band), as well as more posteriorly in the lateral occipital cortex (beta and gamma 1 bands). Medially, clusters were observed in the left precuneus (delta, theta, beta and gamma 1 bands). In the right hemisphere, correlation clusters were found for the delta band in the superior and inferior parietal gyri, and for the beta and gamma 1 bands in the precuneus.

Anticorrelation clusters were found bilaterally in the temporal and frontal/cingulate areas. In the left temporal regions, clusters were found for the theta to beta range in the superior temporal pole and the anterior inferior temporal gyri. On the right side, temporal clusters were restricted to the superior temporal pole for the alpha and beta bands. In the left frontal regions, we detected clusters in the lateral orbitofrontal cortex (delta to gamma 1 range), the inferior frontal gyrus pars orbitalis (alpha and beta bands) and in the insula (beta band). On the right hemisphere, we found clusters in homologous regions: in the lateral orbitofrontal (theta to beta range) and the inferior frontal gyrus pars orbitalis (alpha and beta bands). But we also detected clusters in the inferior frontal gyrus pars triangularis (alpha and beta bands) and opercularis (alpha, beta and gamma 2 bands), and in the rostral middle frontal gyrus (delta to gamma 1 range).

On the left medial face, in the lower frequency bands, anticorrelation clusters were detected in the occipito-temporal regions: in the lingual (delta to alpha range), fusiform (delta and theta bands), parahippocampal (delta to alpha range), entorhinal gyri (delta to alpha range), as well as in the superior (delta to beta range) and inferior (delta to alpha range) temporal poles. In addition, anticorrelation clusters were found in the medial orbitofrontal cortex (theta to gamma 1 range), the caudal anterior cingulate (gamma 2 and 3 bands), the posterior cingulate (gamma 3 band) and the isthmus cingulate (alpha and beta bands). On the medial face of the right hemisphere, the anticorrelation clusters were located in the fusiform (delta to gamma 3 range), the parahippocampal (beta and gamma 1 range) and the superior temporal pole (beta band). In addition, right medial clusters were also found in the posterior cingulate (delta band, beta to gamma 3 range), the isthmus cingulate (theta band) and the paracentral lobule (gamma 3 band).

### Trail Making Test (condition 4): Figure S2

The correlation clusters mainly in the left lateral side, in temporal, parietal and pre and postcentral regions for low frequency bands. These clusters were found in the transverse and superior temporal gyri (delta to beta) as well as in the middle temporal gyrus (theta, alpha). We also found clusters in the pre and post-central gyri for delta, theta and beta bands, and more posteriorly in the supramarginal (theta, alpha) and the inferior parietal gyri (alpha). A few more clusters were detected in on the medial left posterior cingulate (alpha, beta) and in the parahippocampal (delta) gyri. On the right hemisphere, correlation clusters were only found in the lingual and parahippocampal gyri for gamma 2 and 4 bands.

Anticorrelation clusters were fewer and again mostly in the left hemisphere. They were mostly located on the medial left hemisphere: in the superior frontal (delta, theta gamma 3), precuneus (alpha, gamma 2) and paracentral (gamma 3) gyri. A few other anticorrelation clusters were detected in the left caudal superior frontal (theta), precentral (theta) and supramarginal gyri (gamma 2). Right anticorrelation clusters were all for the theta band, in the precentral and inferior frontal (pars opercularis and triangularis) gyri.

### Difference factor clusters between Verbal Fluency and Vocabulary (F2-VOC): Figure S3

Correlation clusters. The F2-VOC correlation clusters were mostly found in the right hemisphere and partly overlapped with the correlation clusters found for Verbal Fluency, but not with those found for Vocabulary. For the lower frequency range (delta to beta) difference factors clusters were found on the right hemisphere from the prefrontal and frontal regions: in the rostral (delta to beta range) and caudal (delta, alpha and beta bands) middle frontal gyrus, lateral orbitofrontal (beta band), inferior frontal pars opercularis (delta to alpha range), and caudal superior frontal gyri (delta, alpha and beta bands). Difference factors clusters were also found on the right hemisphere in the pre-central (delta, alpha and beta bands) and post-central (alpha band) gyri. On the right medial face, difference factors clusters were restricted in the medial orbitofrontal cortex in the delta and the gamma 1 to 3 bands.

The difference factors clusters overlapped on the lateral face with those found for Verbal Fluency in the precentral and caudal middle frontal gyri (theta, alpha and beta bands), as well as in the caudal superior frontal gyrus (alpha band). In these lower frequency bands (alpha to beta), all the other more anterior correlation clusters (rostral middle frontal, rostral superior frontal, lateral orbitofrontal and inferior frontal pars opercularis gyri) were specific to the difference factors. All the other more posterior correlation clusters (superior parietal, supramarginal gyri) were found only for Verbal Fluency. In other words, the central clusters were correlated to the scores of both Verbal Fluency and Vocabulary, but the more posterior clusters were correlated with the scores of Verbal Fluency only, while the more anterior clusters were correlated with the relative advantage in Verbal Fluency as compared to Vocabulary. On the medial faces, a similar pattern was found on both hemispheres. On the right hemisphere, we observed correlation clusters in the lower frequency bands in the medial orbitofrontal gyrus (delta, gamma to gamma 3 bands) for the difference factors, but in the rostral superior frontal gyrus (delta to beta range) and more posteriorly the paracentral lobule (delta, alpha and beta bands) and the precuneus (theta and alpha bands) correlation clusters were specific to Verbal Fluency. On the left medial hemisphere, correlation clusters were found in the rostral superior frontal gyrus (theta to beta range) for both difference factors and Verbal Fluency, but additional prefrontal clusters were found in the medial orbito-frontal (delta to beta bands) and in the caudal anterior cingulate (delta and beta bands) for difference factors alone, while more posterior clusters were detected in the paracentral lobule (delta, alpha and beta bands) and the precuneus (theta band) for Verbal Fluency specifically. With regard to Vocabulary, we observed only small isolated correlation clusters in the superior parietal lobule (delta band) and in the precuneus (beta and gamma 1 bands) on the lateral face of the right hemisphere.

Anticorrelation clusters. The F2-VOC anticorrelation clusters were mostly found on the lateral side of the left hemisphere and largely overlapped with the correlation clusters found for Vocabulary. Difference factors clusters were close to each other around the parieto-temporal junction. In the central and parietal regions, they were found in the post-central gyrus (delta and theta bands), the supramarginal gyrus (delta and theta bands), and in the inferior parietal gyrus (in the delta to beta range). In the temporal regions, they were mostly posterior, in the superior, middle and inferior temporal gyri (from delta to beta bands), and they extended more caudally in the lateral occipital and the fusiform cortex (beta and gamma 1 bands). On the medial face of the left hemisphere an anticorrelation clusters was observed in the isthmus cingulate and the lingual gyrus for the alpha band. The difference factors clusters overlapped on the external face of the left hemisphere with two quite distinct patterns of correlation clusters for Vocabulary (in the temporo-parietal regions) and of anticorrelation clusters for Verbal Fluency (in the temporo-occipital regions). Specifically, they overlapped with correlation clusters for Vocabulary in the post-central (delta and theta bands), supramarginal (delta and theta bands) and inferior parietal gyri (theta to beta bands). In the posterior temporal lobe, in addition, they overlapped with correlation clusters specifically in the superior (delta and theta bands) and middle temporal gyri (delta and theta, beta and gamma 1 bands). On the other hand, the overlap between difference factors and Verbal Fluency correlation clusters was limited to small parts of the middle (delta to beta bands) and inferior (alpha band) temporal gyri, and of the lateral occipital cortex (beta, gamma 2 and 3 bands). On the medial face of the left hemisphere the difference factors cluster overlapped with the anticorrelation cluster for Verbal Fluency in the isthmus cingulate for the alpha band.

### Difference factor clusters between Verbal Fluency and Trail Making Test (F1-TMT): Figure S4.

The F1-MT anticorrelation clusters are associated with a better performance for VFL as compared to TMT. They often overlapped with the correlation clusters we obtained for VFL, but with some differences in location or frequency bands. Like the VFL-correlation clusters, they were found in the right parietal, pre- and postcentral and frontal regions. Specifically, on the right lateral side, we found clusters in the superior parietal (delta to gamma 3 range), in the inferior parietal (theta, alpha, and gamma 1 band) lobe, and also in the supramarginal gyri (delta to alpha bands). More anteriorly, we found clusters in the postcentral and precentral gyri (delta to beta frequency bands, but also gamma 2 and 3 bands (precentral) and gamma 3 band (postcentral). Frontally we observed clusters in the caudal part of the superior frontal (beta bands) as well in the caudal (delta, alpha and beta bands) and rostral middle frontal gyri (delta, alpha to gamma1 bands), as well in the pars opercularis (delta band).

On the left lateral side, the high frequency VFL correlation clusters turned to anticorrelation clusters for the difference factor F1-TMT and included slower frequency bands. Specifically, the F1-TMT anticorrelation clusters in the superior parietal were found from the theta to gamma 3 bands. We found additional clusters in the inferior parietal (gamma 2 and 3 bands). However, the same clusters were found in the supramarginal for the gamma 2 and 3 bands, and in the pre- and postcentral gyrus for the gamma 3 band. In the frontal regions we no longer found clusters in the superior frontal or in the caudal middle frontal gyri, but the cluster in the rostral middle frontal gyri and in the pars opercularis (both gamma 3 band) was retained.

Medial left clusters were found bilaterally in parietal and occipital regions for both the VFL and F1-TMT factors. We detected anticorrelation clusters in the precuneus for the delta to gamma 2 bands bilaterally, while they were limited to theta and alpha bands (left) and theta band (right) for VFL correlation clusters. Additional anticorrelation clusters were found in the cuneus for theta to gamma 2 bands. Somewhat similar to the VFL correlation clusters, we observed paracentral anticorrelation clusters for the delta to theta band in the left hemisphere and for the delta and alpha bands in the right hemisphere. However, the bilateral VFL correlation clusters in the medial superior frontal gyrus (delta to beta bands) turned into a F1-TMT anticorrelation cluster only on the right side for the delta band.

Supplementary Figures


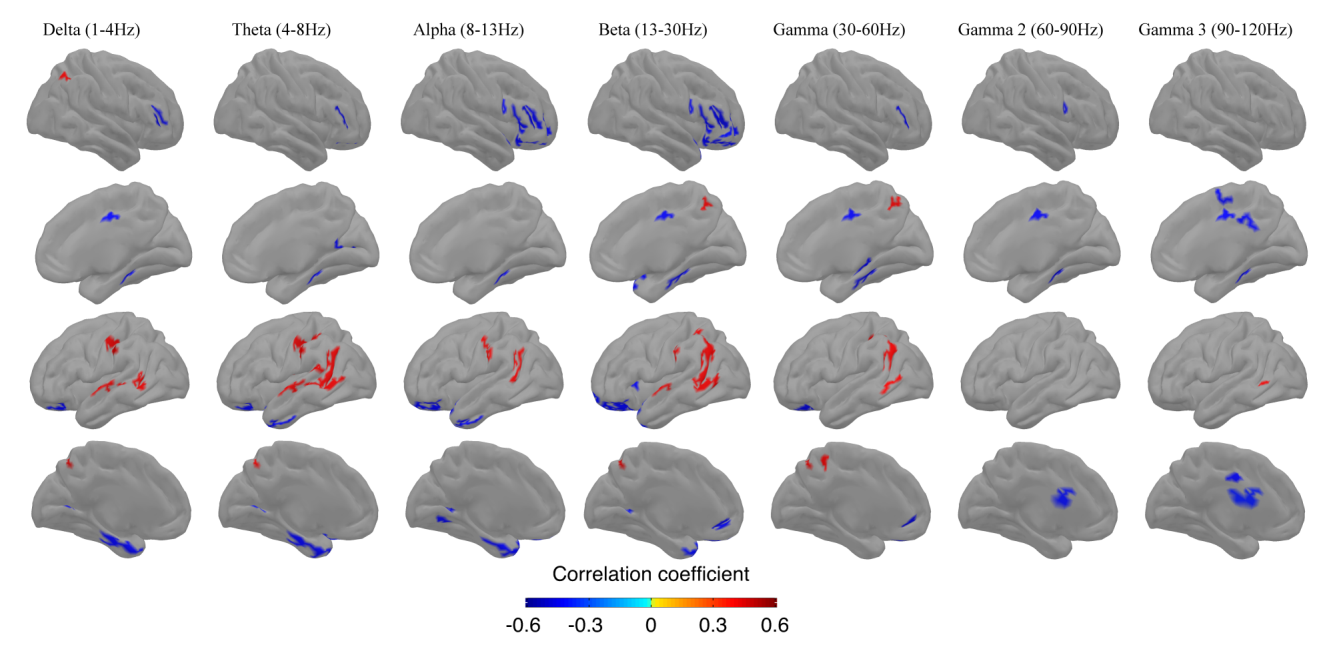


Figure S1: Group analysis (n=28) spatial distribution of clusters with statistically significant correlations (p<.001) in resting MEG source-space power (z-scores across vertices) and neuropsychological performance on the Vocabulary test. The formatting of the figure and statistical significance of the results are identical to those in Fig. 2.


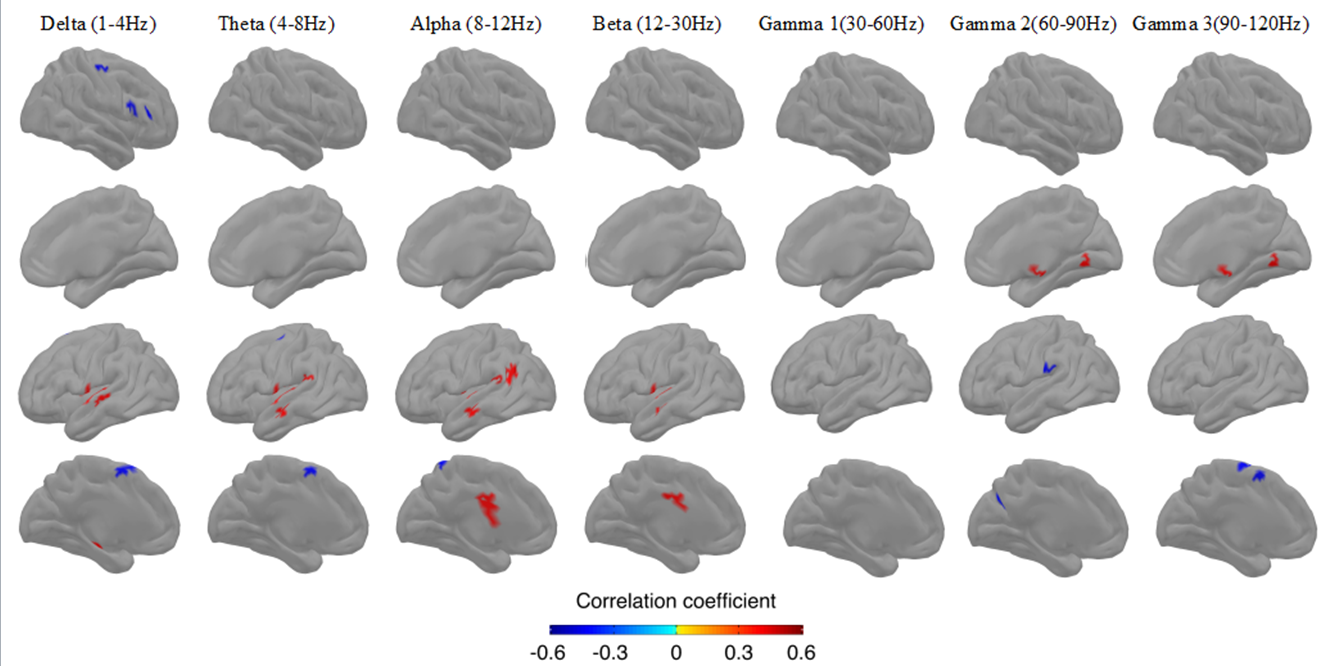


Figure S2: Group analysis (n=28) spatial distribution of clusters with statistically significant correlations (p<.001) in resting MEG source-space power (z-scores across vertices) and neuropsychological performance on the Trail Making Test (Condition 4) test. The formatting of the figure and statistical significance of the results are identical to those in Fig. 2.


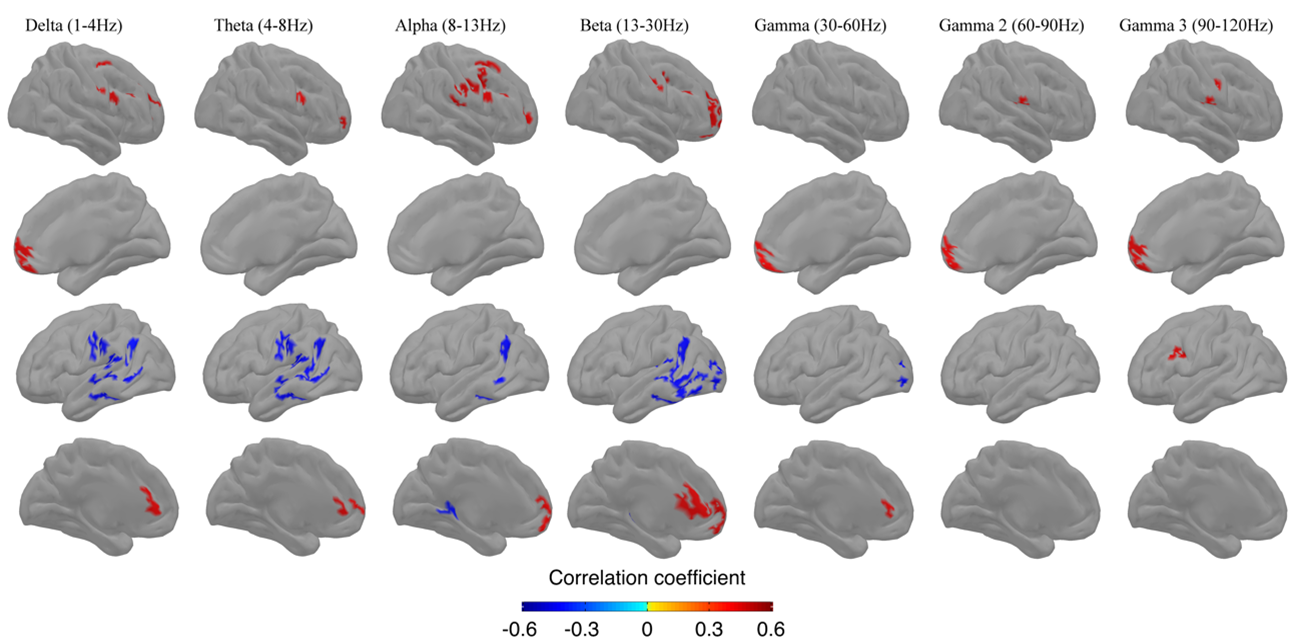


Figure S3: Group analysis (n=28) spatial distribution of clusters with statistically significant correlations (p<.001) in resting MEG source-space power (z-scores across vertices) and F2-VOC individual factor scores. The formatting of the figure and statistical significance of the results are identical to those in Fig. 2.


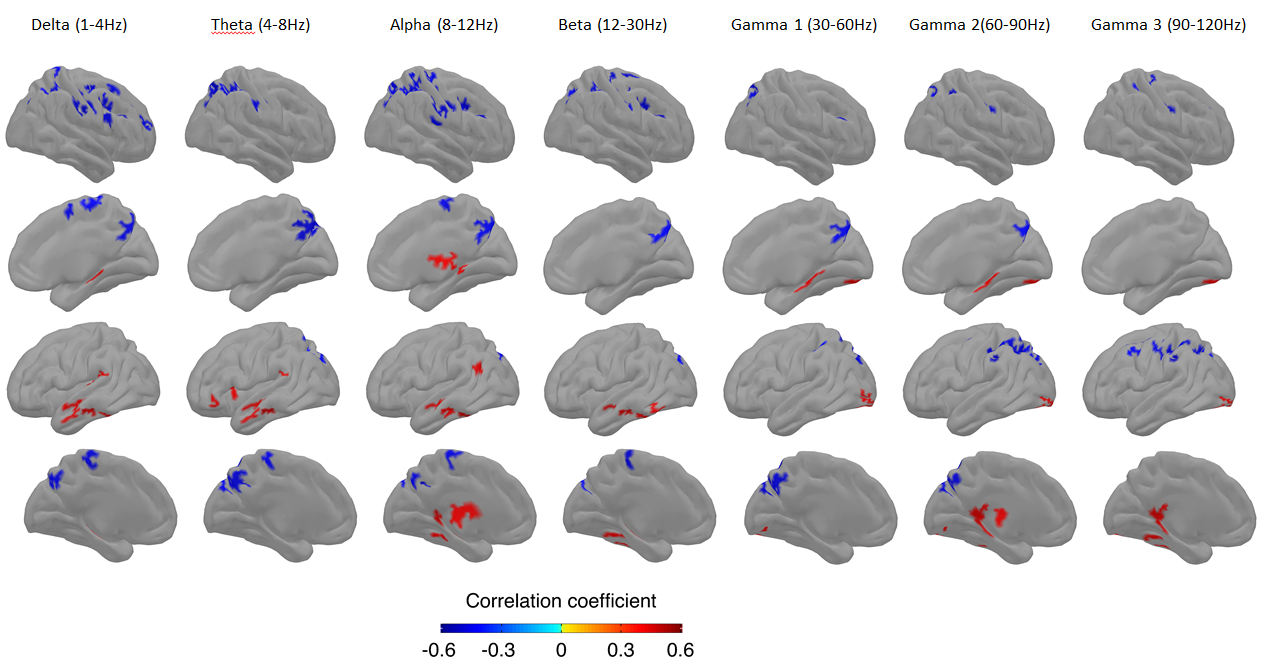


Figure S4: Group analysis (n=28) spatial distribution of clusters with statistically significant correlations (p<.001) between resting MEG source-space power (z-scores across vertices) and F1-TMT individual factor scores. The formatting of the figure and statistical significance of the results are identical to those in Fig. 1.
